# Toxicity of Microplastics and Nanoplastics in Mammalian Systems

**DOI:** 10.3390/ijerph17051509

**Published:** 2020-02-26

**Authors:** Cheryl Qian Ying Yong, Suresh Valiyaveettil, Bor Luen Tang

**Affiliations:** 1 Department of Biochemistry, Yong Loo Lin School of Medicine, National University of Singapore, Singapore 117596, Singapore; cherylyongqy@hotmail.com; 2 Department of Chemistry, National University of Singapore, 3 Science Drive 3, Singapore 117543, Singapore; chmsv@nus.edu.sg; 3 NUS Graduate School for Integrative Sciences and Engineering, National University of Singapore, Singapore 119077, Singapore

**Keywords:** human cells, microplastics, mouse cells, nanoplastics, oxidative stress, toxicants, toxicity

## Abstract

Fragmented or otherwise miniaturized plastic materials in the form of micro- or nanoplastics have been of nagging environmental concern. Perturbation of organismal physiology and behavior by micro- and nanoplastics have been widely documented for marine invertebrates. Some of these effects are also manifested by larger marine vertebrates such as fishes. More recently, possible effects of micro- and nanoplastics on mammalian gut microbiota as well as host cellular and metabolic toxicity have been reported in mouse models. Human exposure to micro- and nanoplastics occurs largely through ingestion, as these are found in food or derived from food packaging, but also in a less well-defined manner though inhalation. The pathophysiological consequences of acute and chronic micro- and nanoplastics exposure in the mammalian system, particularly humans, are yet unclear. In this review, we focus on the recent findings related to the potential toxicity and detrimental effects of micro- and nanoplastics as demonstrated in mouse models as well as human cell lines. The prevailing data suggest that micro- and nanoplastics accumulation in mammalian and human tissues would likely have negative, yet unclear long-term consequences. There is a need for cellular and systemic toxicity due to micro- and nanoplastics to be better illuminated, and the underlying mechanisms defined by further work.

## 1. Introduction

One of the most prominent classes of non-natural products made by humans that has pervaded Earth’s surface environment is plastics, so much so that these durable synthetic organic polymers are heralded as a defining stratigraphic marker for the Anthropocene [1]. Geyer and colleagues recently estimated that 8.3 billion metric tons of virgin plastics have been produced up to the year 2017 [2], and with continuation of current production and waste management practices, about 12 billion tons of plastic waste would be found in landfills and the natural environment by 2050. Plastic wastes are persistent environmental pollutants. Larger pieces of plastic wastes present well-publicized ecological problems in terms of physical entanglement and entrapment [3], physical barriers for food supply [4], and digestive tract congestion. The smaller plastic pieces, particularly their miniaturized forms that are less than 5 mm in size, are generally termed microplastics (MPs) [5]. Plastics that are already small in size to begin with, such as those purposefully manufactured in the form of microbeads in skincare products (primary MPs), or those derived from degradation of larger plastic pieces (secondary MPs), permeate both the terrestrial [6] and the marine [7,8] environments. Plastic particles of less than 1 µm in size are also known as nanoplastics (NPs) [9,10]. These chemically inert MPs/NPs pose significant ecological and health concerns [5] because of their environmental persistence [6,11,12], potential ecotoxicity [13,14], and their ability to act as vectors for chemical pollutants [15,16,17] as well as pathogens [18,19].

Ecotoxicological effects of MPs/NPs on marine phyto/zooplanktons, invertebrates, and plants are widely documented [20,21,22,23,24,25,26,27,28,29,30], and have been recently reviewed [5,31,32,33]. MPs/NPs could also be ingested and accumulated in larger marine fauna by trophic transfer from prey to predator, as demonstrated earlier with invertebrates, such as mussel-consuming crabs [34]. Interesting illustrations of trophic transfer within a lab-simulated food chain were shown by Mattsson and colleagues, where 53-nm polystyrene (PS) particles could be transferred from algae to the zooplankter *Daphnia magna*, and then to a freshwater fish [35]. Likewise, An and colleagues demonstrated trophic transfer of NPs from algae to *Daphnia*, then to a secondary consumer fish, and finally to an end consumer fish [36]. On the other hand, organic pollutants could be adsorbed onto MPs/NPs [37,38,39,40,41] and there is evidence that this could potentially enhance their effective uptake and toxicity [42,43,44,45]. Likewise, MPs/NPs are known to interact with metallic toxicants such as Cadmium [46,47,48], Mercury [49], and other toxic trace elements [50], and could potentially serve as vectors for pollutant transfer to living organisms.

The effects of MPs/NPs on mammalian cells and tissues, particularly humans, have remained rather unclear [51,52]. While plastics are generally perceived to pose minimum risk to human, several recent scientific findings, picked up by the popular press, have heightened the worry of possible tissues penetrance and adverse effects of MPs/NPs due to their small sizes. Humans could accumulate MPs/NPs from different food sources [53,54] as well as drinking water [55,56]. Plastic water containers [57,58] and plastic teabags [59] are, perhaps unsurprisingly, common sources for human ingested MPs/NPs. MPs/NPs could also be taken up by inhalation [60]. MPs/NPs have also been detected in human stool samples [61], an indication that the quantity taken in is significantly large. A recent World Health Organization’s (WHO) report on “Microplastic in drinking water” indicates that there is not yet proof of harm, but it also calls for more research to be carried out [62]. 

Could environmental MPs/NPs gain access to cells and tissues and be harmful to humans? Although ecotoxicology data with marine invertebrate indicate that this is so, more barriers and obstacles would likely be encountered by MPs/NPs in order to gain access to cells and tissues of vertebrates compared to simpler invertebrates. Here, we review current results on how MPs/NPs might affect humans by scrutinizing studies done to date on mammalian (mouse) models and human cells. We begin with a quick survey of MP/NP feeding studies done on marine vertebrates, focusing on fishes. A meta-analysis on the effect of MP exposure on fish has been reported by Foley and colleagues in 2018 [63] and the field has also been recently reviewed [64], but several newer reports have now appeared. This quick look would allow some comparison of findings in more ecologically relevant settings with that of laboratory experiments with mice and human cells.

## 2. Toxicity of MPs/NPs in Fishes

Table 1 provides a non-exhaustive summary of recent studies where MP/NP feeding experiments have documented some degree of toxicological or pathological effect observed on fishes. Those that have shown some significant effect are included in this summary, while those that have reported little or no effects are not. The MPs/NPs used in the studies listed in Table 1 are largely polystyrene (PS) or polyethylene (PE) based. An important general phenomenon to note is that toxicological responses typically arise from smaller plastic particles. Larger PS particles at around 100 µm or above were shown not to have any significant effect in a number of studies [65,66,67]. MP/NP feeding can result in behavioral abnormalities in terms of feeding and movement of adults and larvae [35,68,69,70,71,72,73,74], as well as reproduction in adults [75,76,77]. There is evidence of mother–offspring transfer of NPs [78], and that prenatal exposure of MPs affected early development of the neonates [77].

In many cases, MPs/NPs were found accumulated in larvae or adult gut [77,79,80,81,82,83], and in some cases in gill and liver [79]. Histopathology is most prominently observed for these tissues as well [74,75,77,79,83,84,85]. For the gut, pathological manifestations of MP/NP toxicity include documented changes in gut biomarkers related to epithelial barrier integrity, inflammation, and oxidative stress [83,86], as well as changes in gut microbiota [82,83,86]. In the case of liver, changes in metabolites, key metabolic enzymes, and oxidative stress-induced enzymes occur [49,74,79,81,85,87]. MPs/NPs could be internalized [88], and then cause detectable biomarker changes in blood cells [47,88,89]. In rarer cases, MPs/NPs have also been found in fish brain [68,81], and caused changes in brain appearances [35,68] or showed significantly inhibited acetylcholinesterase (AChE) activity [49,81]. NPs taken up by embryos and larvae have been documented to migrate to various tissues throughout development [70].

In line with MPs/NPs activity as carriers or vectors of environmental contaminants, studies using fish cell lines have revealed that, while pristine plastics show no toxicity, those sampled from different islands around the world do [96], and so do those that have been mixed with human pharmaceuticals [97]. MPs/NPs were shown to modulate the toxicities a range of pollutants/toxicants, including phenanthrene [75], mercury [49], cadmium [46,47,48], polychlorinated biphenyls (PCBs) [98], gold ions [99], and the antibiotic roxithromycin [100], in fishes. However, adsorption of toxicants by the plastics could also potentially lower their toxicity, and such is the case for a complex mixture of polycyclic aromatic hydrocarbons (PAHs) [101]. Beyond MP/NP feeding experiments in a laboratory setting, a sampling of wild fishes consumed by humans have indicated that those with MPs found in the gut and other tissues had significantly higher lipid peroxidation levels in the brain, gills, and dorsal muscle and increased brain AChE activity compared to fishes with no MP found in their tissues [95]. These correlations are strongly suggestive of MP/NP uptake being a general stress factor for marine vertebrates.

Overall, despite a large variance in MPs/NPs used, fish models tested, and toxicity parameters examined, there is ample evidence of concentration-dependent acute toxicity as well as chronic effects. Furthermore, environmental toxicants adsorbed onto MPs/NPs would likely change the plastics’ toxicity profiles, often with an enhancement of toxicant uptake or an increase in their bioavailability. 

## 3. Toxicity of MPs/NPs in Mouse Models

In the past three years, a good number of studies have examined the effect of pristine MPs/NPs in mammalian models (largely mouse). These studies are summarized in Table 2 and are broadly recapped below. In mice, ingested MPs/NPs could be found in the gut [102,103,104,105], liver [102,103,105], and kidney [102,105]. Pathological changes to the gut include a reduction in mucus secretion [90], gut barrier dysfunction [104,106], intestinal inflammation [107] and gut microbiota dysbiosis [90,104,106,107]. Liver pathologies documented include inflammation and lipid accumulation or lipid profile changes [90,102,106], as well as changes in the markers of lipid metabolism [90,105,108]. Other metabolic problems noted by omics-type analyses include disorders in energy metabolism [102,105] and bile acid metabolism [104]. On the other hand, a study with mice fed with PS MPs did not reveal any histologically detectable lesions or significant inflammatory responses [109]. A neurobehavioral study on rat fed with NPs also did not detect any significant behavioral changes or abnormality [110].

Recently, Luo and colleagues documented that maternal exposure to PS during gestation causes metabolic disorders in the offspring [106,108]. As in fishes, MPs aggravated the toxicity of an environmental toxicant, organophosphorus flame retardants (OPFRs) [103]. Taken together, the works in mice feeding experiments recapitulated some of MPs/NPs’ acute toxicity observed in fish feeding experiments. Such observed toxicities correlated with plastic size [111], concentration [112], and cellular/tissue uptake and accumulation. In general, the degree of MP/NP toxicity observed in mice is less severe than that observed in fishes. One possible reason is that fishes have multiple routes for plastic uptake and accumulation (gut and gills), whereas mice feeding experiments limit uptake through the gastrointestinal route.

## 4. Toxicity of MPs/NPs in Human Cells

Could MPs/NPs affect human cells and tissues? There is obviously a lack of toxicity data for humans in vivo at the moment. Several studies have, however, looked at the effect of pristine MPs/NPs on human cells in culture. These works are summarized in Table 3. Not surprisingly, a few of these studies, despite documenting some degree of cellular uptake, found signs of cellular toxicity either absent or insignificant except at very high concentrations of MPs/NPs [109,113,114]. In one case, where polyethylene terephthalate (PET) NPs generated by laser ablation were tested on the human gut adenocarcinoma epithelial line Caco-2, the authors noted a propensity for NP uptakes and crossing of a Caco-2 cells-based intestinal barrier model [113].

A few other studies have documented some degree of cellular toxicity or pathological effect in a range of human cell lines. Prietl and colleagues showed that 20 nm PS NPs are taken up easily by human monocytic cells and are significantly cytotoxic. Larger (100 and 1000 nm) NPs stimulated the secretion of cytokines such as IL-6 and IL-8 from monocytes and macrophages, and could, interestingly, induce a measurable degree respiratory burst in monocytes [115]. Schirinzi and colleagues documented low but measurable degree of reactive oxygen species (ROS) production and induction of cytotoxicity by MPs in T98G and HeLa cells [116]. Wu and colleagues also worked with Caco-2 cells, and reported that, while a low degree of toxicity was observed for PS NPs (at 0.1 and 5 µm), they induced mitochondrial depolarization and inhibited the activity of the toxicant efflux pump, ATP-binding cassette (ABC) transporter, with the latter resulting in increased arsenic toxicity [117]. Hwang et al. worked with a number of cell types of human and mouse origin, and documented cytotoxicity associated with 20 µm PP MPs at high concentrations and ROS induction [118]. The MPs also measurably induced pro-inflammatory cytokines IL-6 and TNF-α from human peripheral blood mononuclear cells (PBMCs), and increased histamine release from mast cell lines [118]. Poma and colleagues found that 100 nm PS NPs stimulated ROS production and induced genotoxic stress and DNA damage as measured with the cytokinesis-block micronucleus (CBMN) assay [119].

Owing to the presence of large amounts of plastic particles in air, terrestrial animals are also exposed to MPs/NPs via inhalation. In this connection, Dong and colleagues found that PS MPs produced some cytotoxic effects, oxidative stress, and inflammatory responses in human lung epithelial cells, and are disruptive of the epithelial cell layer, at least in vitro [120]. Two other groups have recently checked the toxicity of NPs in lung [121] and bronchial [122] cells. Xu and colleagues found that PS NPs (25 and 70 nm) impaired viability, induced cell cycle arrest, and upregulated nuclear factor (NF)-κB as well as some pro-inflammatory cytokines in the human alveolar epithelial line A549 [121]. On the other hand, Lim and colleagues noted that PS NPs are only cytotoxic at high concentrations but induced metabolic changes and endoplasmic reticulum (ER) stress in a human bronchial epithelial cell line [122].

Overall, the experiments with pristine MPs/NPs on human cells reported thus far did not indicate severe cytotoxic or cytostatic effects, but did demonstrate a potential for low to moderate negative effects depending on the cell type, MP/NP sizes, and degree of cellular uptake. Two general and prominently observed phenomena appear to be ROS production and pro-inflammatory responses. In the paragraphs below, we explore how these toxic effects may occur.

## 5. Mechanisms Underlying MPs/NPs’ Acute or Chronic Toxicity in Mammalian Cells 

In general, extremely high concentration of MPs/NPs are indeed cytotoxic. Cell death could occur via necrotic plasma membrane rupture or some form of programmed cell death. An important point to note on the former, rather non-specific mode of death is the surfactant molecules that are typically associated with most MP/NP preparations. At high concentrations, these would be disruptive to the lipid bilayer of the plasma membrane (PM). Even at moderate levels, these could disrupt important cellular surface structures such as proteoglycans and other extracellular matrix components or hinder cellular signaling processes that require extracellular ligand and cell surface receptor interactions. Therefore, cellular physiology would be affected to varying degrees by plastic associated surfactants, and the documented changes in various transcripts in cells could also be due to this and other processes/factors described below.

The smaller NPs in particular could be taken up with some ease depending on the cell type via endocytosis [123,124]. Endocytosed NPs present a problem for several reasons. Firstly, they could, as per the plasma membrane discussed above, potentially permeabilize the endosomal membranes if present at high concentrations. If this happens, the NPs released into the cytosol could potentially interact with and affect important organelles such as the mitochondria or the nucleus, as well as cellular processes such as mitotic spindle formation and chromosomal migration during cell division. Secondly, MPs/NPs would likely interfere with the trafficking of transport carriers in the cell along the exocytic pathway [125,126], and as such would potentially inhibit the cell surface expression of important signaling receptors or membrane transporters. Thirdly, they are likely to perturb endosomal membrane traffic on which many important cellular processes are dependent, including surface protein turnover and signaling attenuation, as well as retrograde signaling from endosomal compartments. It is unclear if NPs could themselves ever be subjected to inter-compartmental transport efficiently in the endosomal pathway. Even if the NPs could eventually end up in the lysosome, they are unlikely to be readily digested. The accumulation of NPs in late endosome or lysosomes would perturb the degradative functions of these organelles and importantly impair the critical cellular membrane turnover process of macroautophagy [122]. An impairment of autophagic clearance could potentially lead to positive feedback processes that culminate in autophagic cell death. On the other hand, internalized MPs/NPs may also stimulate autophagy. Metallic nanoparticles are known to modulate autophagy [127], and MPs/NPs may speculatively do likewise.

**Figure 1:**
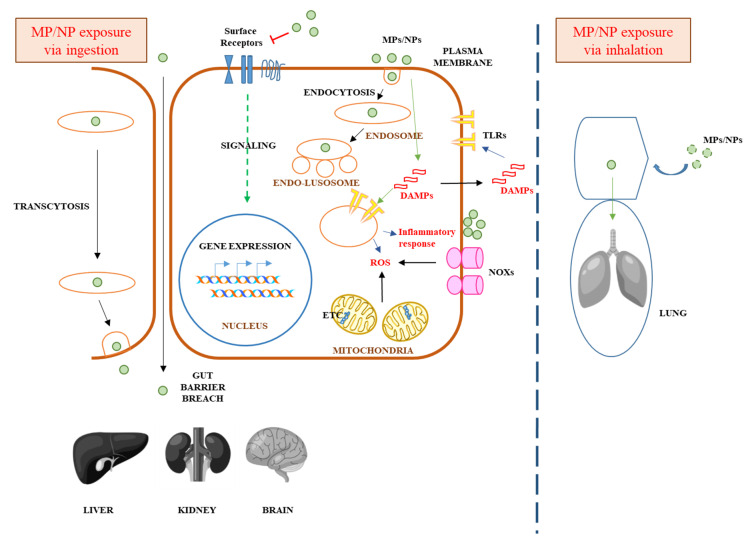
A schematic diagram illustrating potential (speculative at the moment) cellular mechanisms of MP/NP toxicity. MPs/NPs can be taken up through ingestion and inhalation. MPs/NPs could damage the plasma membrane and impair the gut barrier (left). These could also perturb signaling of cell surface receptors, and alter gene expression in the nucleus. Endocytosed MPs/NPs could also perturb the endocytic pathway function and compromise the endosomal membranes. Stresses arising from the above could activate the cellular innate immune system, with endogenous and secreted damage-associated molecular patterns (DAMP) inducing the innate immunity-mediating toll-like receptors (TLRs). Stresses could induce ROS production from the NADP oxidases (NOXs). Mitochondrial impairment, either by MPs/NPs from endosomes or in response to stresses, could also produce more ROS through impairment in the efficiency of electron transport chain (ETC) processes. MPs/NPs gain access into the circulation if the gut–vascular barrier is compromised or it may speculatively occur by transcytosis, thus reaching other organs. The lung probably has a more direct access to airborne MPs/NPs (right).

At the very least, these processes would constitute a form of cellular stress. Stresses at the PM and the endo-lysosomes would trigger cellular stress responses. In work done with species of the fresh water flea *Daphnia*, PS NPs exposure affected growth and reproduction [128], and interestingly resulted in the elevation of AMP activated protein kinase (AMPK), which is an indication of stress response [129]. Perhaps a more general associated phenomenon with regards to cellular stress response appears to be the production of ROS, which was in fact recently identified as the molecular initiating event (MIE) by adverse outcome pathways analysis of reports in the field [130]. ROS production in cells occurs in two general ways: from the mitochondrial electron transport chain (ETC) during routine aerobic respiration or via the oxidative bursts of NADPH oxidases (NOXs) [131]. An increase in ROS from the former could result from mitochondrial function impairment, while the latter is normally a consequence of bacterial invasion, as NOXs are activated by bacterial products and cytokines. All cells are endowed with an evolutionarily conserved innate immunity mechanism, typically functioning against invasion of pathogens or exposure to xenobiotics [132]. However, the components of the innate immune system, such as the Toll-like receptors (TLRs), could also respond to a set of endogenous or secreted molecules collectively known as alarmins, or damage-associated molecular patterns (DAMP) [133,134], and the outcome is what is termed sterile-inflammation, i.e. inflammatory responses without pathogenic infection [135]. In the body, pro-inflammatory cytokines released from such localized inflammations would attract circulating immune cells, and this could worsen the local inflammation, and cause cell and tissue death. NPs has indeed been shown to act as stressors to the innate immune system of fish [88], and this is likely also the case for mammalian (including human) cells. The cellular and tissue invasion and general pathological mechanism of MPs/NPs in mammalian cells is summarized below in Figure 1.

## 6. MPs/NPs’ Potential Systemic Effect in Humans

MPs/NPs are expected to reach the human gut through consumption of contaminated food materials. Undigested MPs would be largely excreted though fecal matter, but smaller NPs could potential enter the circulation. Ingested MPs/NPs would first encounter the intestinal epithelium. Only unrealistically high concentration of plastics, or those carrying adsorbed toxicants, would likely cause acute impairment of viability and inflammation of the gut lining [51]. However, the effect of persistent presence of inefficiently cleared MPs/NPs in the gut is yet unknown. Gut pathology resulting from MPs/NPs has been widely documented in fishes. The mouse experiments have provided some clear illustration of the consequences of gut toxicity. Should this happen, the gut–vascular barrier could be impaired and MPs/NPs could enter the circulation, where these could gain access to the liver via the portal vein. That this is possible was demonstrated in some of the mouse models [102,103,105]. Long-term accumulation of MPs/NPs in liver tissues and chronic inflammation could lead to liver diseases and metabolic problems. On the other hand, accumulation of MPs/NPs in lung tissues could potentially result in chronic pulmonary disorders. Furthermore, the presence of NPs in brain tissues has been demonstrated in a fish model, as discussed above [68]. It should, however, be noted that it remains to be shown if the MPs/NPs could in fact be found in the brain of experimental mice or human brain samples. For that matter, no cell or tissue accumulation, pathology, or metabolic impairment due to MPs/NPs has been clearly demonstrated for humans to date. 

One of the more common findings in the mice studies is gut microbiota dysbiosis [90,104,106,107]. Changes in gut microbiome could result in gustatory dysfunction, thus perturbing physiological homeostasis in general. More importantly, gut microbiota changes have been linked to a range of chronic diseases of other organs, including disease of the kidney [136], cardiovascular system [137], inflammation, and cancer [138], as well as neurological disorders [91,139]. With regards to the latter, gut microbiota dysbiosis could in fact be one of the underlying reasons for behavioral changes in larger animals treated with MPs/NPs.

It has also been reported that blood proteins such as albumin and globulin interact with NPs to form protein–plastic complexes [140,141]. Such aggregated protein–plastic complexes, if present in large quantities, could potentially lead to blockage of blood vessels. In addition, while loading of red blood cells (RBCs) with NPs at a low 1:50 ratio did not affect functions of RBCs, a 10–50-fold higher loading showed RBC damages induced by mechanical, osmotic, and oxidative stresses [142]. However, it is difficult to envisage a large acute accumulation of NPs to occur in the human circulation under natural conditions.

## 7. Conclusions

MPs/NPs have pervaded the environment and human’s exposure and cumulative uptake of these plastics would only increase over time. Currently, it appears that any worries of acute toxicity or severe long-term effect that would lead to significantly enhanced morbidity or mortality are unfounded. However, we still know very little about how MPs/NPs from the environment, be it from a seafood meal or the plastic bottled drink, would affect human health. Clearly, much more research, in terms of both cellular and tissue level pathological mechanisms, as well as on the long-term effects of tissue/organ accumulation, is needed. Plans and collaborative attempts between ecologists and epidemiologists to study bioaccumulation of MPs/NPs in humans via the food chain in various geographical locales would also be necessary.

## Figures and Tables

**Table 1:** A summary of notable toxicological and/or pathological findings associated with MPs/NPs in fishes. PA, polyamide; PS, polystyrene; PE, polyethylene; PC, polycarbonate; PP, polypropylene; PVC, polyvinylchloride; NPs, nanoplastics (<1 µm); MPs, microplastics.

Fishes	Properties of MPs/NPs Used	Tissue Accumulation/ Invasion or Cellular Uptake	Notes on Toxicological, Pathological, or Behavioral Observations	References
Crucian Carp (*Carassius carassius*)	24 and 27 nm polystyrene (PS) nanoparticles (NPs) (to fish through an aquatic food chain, from algae through *Daphnia*)	Trophic transfer to fish from algae through *Daphnia*	• Defects in feeding and shoaling behavior • Defects in metabolism • Changes in brain appearance and weight	Mattsson et al., 2015 [35]
Zebrafish (*Danio rerio*)	Virgin PS microplastic beads (5 µm) + cadmium (Cd)		• Increased Cd accumulation in livers, guts, and gills • Enhanced Cd toxicity • combined exposure caused oxidative damage and inflammation in tissues	Lu et al., 2018 [90]
European seabass (*Dicentrarchus labrax*)	Fluorescence red polymer microspheres, (1–5 μm) and mercury individually and in combination		• Inhibition of brain acetylcholinesterase (AChE) activity and increase lipid oxidation in brain and muscle • Changes in activity of metabolic enzymes • Interactions and influences on mercury bioaccumulation	Barboza et al., 2018 [49]
Crucian Carp (*Carassius carassius*)	Amino-modified positively charged PS nanoparticles (52 nm)	Trophic transfer to fish from algae through *Daphnia*. Nanoparticles found in fish brain	• Changes in feeding time • Changes in brain morphology (gyri sizes)	Mattsson et al., 2017 [68]
Zebrafish (*Danio rerio*)	PS NPs (50 nm, 1 mg/L)	Accumulation in zebrafish larvae	• Inhibited of larvae locomotion • Inhibited acetylcholinesterase activity • Upregulation of cytoskeletal markers	Chen et al., 2017 [69]
African catfish (*Clarias gariepinus*)	Virgin (50 or 500 µg/L) or phenanthrene-loaded (10 or 100 µg/L) low-density polyethylene (LDPE) fragments		• Liver and gill histopathology • Changes in blood biochemistry • Changes in the expression of reproductive axis genes	Karami et al., 2016 [75]
Medaka (*Oryzias melastigma*)	PS microspheres (10–11 μm, 0.758 ± 0.217 × 10^5^ particles/L)	Microplastics observed in observed in digestive tracts of larvae and dissected intestine of adults	• Increased mortality and decrease in average lengths and weights of larvae and adult fishes • Significant decrease in egg production by females	Cong et al., 2019 [76]
Zebrafish (*Danio rerio*)	PS NPs (mean 51 nm)	Uptake of the nanoparticles by embryos and larvae. Migrated to the gastrointestinal tract, gallbladder, liver, pancreas, heart, and brain throughout development	• Decreased heart rate • Altered larval behavior (swimming hypoactivity in exposed larvae) • Maternal-offspring transfer of PS nanoparticles • Delay/defect in swim bladder inflation by exposed F1 larvae	Pitt et al., 2018 [70,78]
Zebrafish (*Danio rerio*)	PS microspheres (70 nm, 5 μm, and 20 μm, 20 mg/L)	Accumulation in gills, gut, and liver (only the 5 μm particles)	• Liver histopathology (signs of inflammation and lipid accumulation) • Elevation of anti-oxidative stress enzymes • Changes in liver metabolomics profile	Lu et al., 2016 [79]
Zebrafish (*Danio rerio*)	PS MPs (10–45 µm, 20 mg/L)	Ingested microplastics observed in larvae gut	• Significant changes in transcriptome of zebrafish larvae after 2 days exposure • Downregulation of genes involved with neural development and function • Changes in genes associated with metabolism	LeMoine et al., 2018 [80]
Red tilapia (*Oreochromis niloticus*)	PS NPs (0.1 µm, at 1, 10, and 100 μg/L)	PS MPs found in gut and gills and to a lesser extent, liver and brain	• Inhibition of brain acetylcholinesterase (AChE) activity • Changes in liver enzyme markers	Ding et al., 2018 [81]
Zebrafish (*Danio rerio*)	Fluorescent and virgin PS MPs (5 and 50 µm)	Ingested microplastics observed in gut of larvae	• Changes in larval gut microbiota • Metabolomic alterations • Changes in the expression of genes associated with glucose and lipid metabolism • Significant reduction in the antioxidant GSH and the enzyme catalase	Wan et al., 2019 [82]
Zebrafish (*Danio rerio*)	PS microplastic beads (5-μm beads; 50 μg/L and 500 μg/L)	Accumulation of microplastics in zebrafish gut	• Induction of inflammation and oxidative stress of adult zebrafish gut • Significant alterations in the metabolome and microbiome of adult zebrafish gut. Alterations were associated with oxidative stress, inflammation, and lipid metabolism	Qiao et al., 2019 [83]
Zebrafish (*Danio rerio*)	Polyamides (PA), polyethylene (PE), polypropylene (PP), polyvinyl chloride (PVC) (~70 µm) and PS (0.1, 1, and 5 µm) particles		• Intestinal damage of adult fish gut	Lei et al., 2018 [84]
Fathead minnow (*Pimephales promelas*)	PS (41.0 nm) and polycarbonate (PC) (158.7 nm) NPs	Neutrophil phagocytosis of PS nanoparticles.	• significant increases in innate immune response (in terms of degranulation of primary granules and neutrophil extracellular trap release)	Greven et al., 2016 [88]
Gilthead seabream (*Sparus aurata*) and European sea bass (*Dicentrarchus labrax*)	Virgin polyvinylchloride (PVC) and polyethylene (PE) (40–150 μm)		• Increased oxidative burst of in leukocytes of *Sparus aurata* • Upregulation of the redox regulator Nrf2 in leukocytes of *Sparus aurata*	Espinosa et al., 2018 [89]
Carp (*Cyprinus carpio*)	MPs from a face and body scrub, mainly PE (250 and 500 μg/L), alone and + Cd		• Changes in plasma levels of various metabolic enzymes and immune markers • Combination of MP and Cd increased Cd toxicity	Banaee et al., 2019 [47]
Black rockfish (*Sebastes schlegelii*)	PS MP/NPs (0.5 and 15 μm at 190 μg/L)		• Changes in behavior, including reduction in fish swimming speed and range of movement • Increased oxygen consumption and ammonia excretion, reduction of growth and energy reserve, with microparticles having greater effect than nanoparticles	Yin et al., 2019 [71]
Zebrafish (*Danio rerio*)	PE MPs (10–600 μm at 2 mg/L)	MPs accumulation in gill and intestine	• Abnormal behaviors, including erratic movement, seizures, and morphological changes associated with MP feeding of adult fishes • Upregulation of intestinal Cytochrome P450 gene (*cyp 1a*) and liver vitellogenin 1	Mak et al., 2019 [72]
Medaka (*Oryzias melastigma*)	PS nanoparticles (10 μm at 2–200 μg/L)	MPs accumulation in gill, intestine, and liver	• Oxidative stress and structural damages in tissues with MP accumulation • Reproductive endocrine disruption in a sex-dependent manner. • Prenatal exposure to MPs affected the early development of offspring	Wang et al., 2019 [77]
Zebrafish (*Danio rerio*)	PS nanoplastics, 25 nm	NP accumulation in intestine, pancreas, and gallbladder of exposed larvae	• Disruption of glucose homeostasis • Increase cortisol levels and hyperactivity	Brun et al., 2019 [87]
Zebrafish (*Danio rerio*)	PS and PE NPs (with size distribution indicated as 90% < 90 µm; 50% < 50 µm; 10% < 25 µm)		• Alterations in intestinal mucosa and gill epithelium with higher neutrophil infiltration • Changes in the expression of immune system genes, down-regulation of genes correlated with epithelium integrity and lipid metabolism • Changes in daily activity pattern	Limonta et al., 2019 [91]
Japanese Medaka *(Tigriopus japonicas)*	PS MP/NPs, 50 nm and 10 μm		• Increase in ROS with corresponding changes in GSH and antioxidant enzyme activities	Choi et al., 2019 [92]
*Oryzias latipes*	PS MPs, 10 μm	MP accumulation in gill and gut	• Dose-dependent decreases in egg number in mature females • Swollen enterocytes and histological alterations of buccal cavity, head kidney, and spleen	Zhu et al., 2019 [93]
Zebrafish (*Danio rerio*)	PE MPs, 38.26 ± 15.64 µm		• MPs induced significant changes in morphometric parameters of larvae • MPs cause lower larval survival rate after egg hatching.	Malafaia et al., 2019 [94]
Japanese Medaka *(Tigriopus japonicas)*	Environmental MP samples collected from beaches		• Larvae ingestion of MPs decreased viability, decreased head/body ratios, increased Ethoxyresorufin-O-deethylase (EROD) activity, DNA breaks and altered swimming behavior • Juveniles exhibited no symptoms except for increase in DNA breaks	Pannetier et al., 2020 [73]
Goldfish (*Carassius auratus*)	PS MP/NPs, 70 nm and 5 µm, at 10, 100 and 1000 μg/L		• MP/NPs inhibit fish larvae growth at high levels, increased larvae heart rate and decreased swimming speed • Observations of histopathological changes to intestine, liber and gill, and damages to skin and muscle • MP/NPs elevated oxidative stress markers and related enzymes	Yang et al., 2020 [74]
Carp (*Cyprinus carpio*)	PVC MPs, ~100–200 μm, at 45.55, 91.1, and 136.65 μg/L		• MPs reduced weight and body length of carp larvae • Histopathological changes in liver • Elevated oxidative stress and related enzyme activities	Xia et al., 2020 [85]
**Non-laboratory feeding observations**				
Wild fishes (*Dicentrachus labrax*, *Trachurus trachurus*, *Scomber colias*) sampled from North East Atlantic Ocean	MPs found in 49% of fishes	MPs found in gastrointestinal tract, gills. and dorsal muscle	• Fishes with MP have significantly higher lipid peroxidation levels in the brain, gills. and dorsal muscle and increased brain acetylcholinesterase activity	Barboza et al., 2019 [95]

**Table 2:** A summary of notable toxicological and/or pathological findings associated with MPs/NPs in mouse. PS, polystyrene; PE, polyethylene; PVC, polyvinylchloride; NPs, nanoplastics (<1 µm); MPs, microplastics.

Properties of MPs/NPs Used	Tissue Accumulation/ Invasion or Cellular Uptake	Notes on Toxicological, Pathological, or Behavioral Observations	References
**Significant Toxicity/Pathology**			
Polystyrene (PS) microspheres 5 μm and 20, 0.01–0.5 mg/day	Accumulation in gut, liver, and kidney	• Signs of inflammation and lipid accumulation in liver • Altered lipid profile and impairment of energy metabolism (reduction in ATP levels) • Increased liver oxidative stress markers, decreased acetylcholinesterase	Deng et al., 2017 [102]
PS particles (0.5 and 50 µm)		• Decreased in body, liver, and lipid weights • Decreased mucus secretion in the gut • Alteration in gut microbiota • Changes in hepatic lipid profile and expression of some genes related to lipid metabolism	Lu et al., 2018 [90]
PS and PE beads (0.5–1.0 μm) + organophosphorus flame retardants (OPFRs)	PS and PE beads detectable in gut and liver	• MPs enhanced OPFR-induced oxidative stress, neurotoxicity, and metabolic disorder compared to OFPR alone.	Deng et al., 2018 [103]
PS particles (5 µm, 100 and 1000 µg/L)	Accumulation in mouse gut	• Caused intestinal barrier dysfunction • Induced gut microbiota dysbiosis • Induced bile acids metabolism disorder	Jin et al., 2018 [104]
PS particles (5 and 20 µm)	Accumulation in mouse gut, liver, and kidney	• Toxicokinetic/toxicodynamic (TBTK/TD) modeling of organ-bioaccumulation and biomarker responses • Changes in oxidative stress markers and those of energy and lipid metabolism	Yang et al., 2019 [105]
PS MPs (5 µm)		• Noticeable liver histopathology and altered serum and hepatic markers • Changes in transcript of genes related to glycolipid metabolism • Metabolic disorder associated with gut microbiota dysbiosis and gut barrier dysfunction. • Maternal MPs exposure resulted in intergenerational effects and caused long-term metabolic consequences in the F1 and F2 generations.	Luo et al., 2019 [106]
PS MPs (0.5 and 5 µm)		• MP exposure caused changes in serum and liver metabolic markers • Maternal MPs exposure caused fatty acid metabolic disorder in the F1 offspring	Luo et al., 2019 [108]
PS MPs (10–150 μm)		• MP exposure affected composition and diversity of gut microbiota • increased the secretion of IL-1α in serum, and decreased the Th17 and Treg cells among CD4+ cells • High-concentration of MPs induced inflammation of the small intestine	Li et al., 2019 [107]
**No Effect or Insignificant Effect**			
PS particles (25 and 50 nm)		• No significantly measurable neurobehavioral consequences in rats	Rafiee et al., 2018 [110]
PS particles (1, 4 and 10 µm)		• No significant effect on body/organ weight and no pathological signs by histological examination • Reporter analyses did not reveal evidence for the occurrence of inflammation and/or oxidative stress • Very low number of particles taken up by intestinal tissue	Stock et al., 2019 [109]

**Table 3:** A summary of notable toxicological findings associated with MPs/NPs in human cells. PS, polystyrene; PE, polyethylene; PVC, polyvinylchloride; NPs, nanoplastics (<1 µm); MPs, microplastics.

Human Cell Models	Properties of MPs/NPs Used	Cellular Uptake	Notes on Toxicological Observations	References
**Significant Toxicity**				
Human Peripheral blood monocytic cells (PBMCs) U937 (human monocytic cell line) THP-1 (human monocytic cell line) DMBM-2 (mouse macrophage cell line)	Carboxylated PS NPs (20–1000 nm)	20 nm nanoparticles taken up passively, while larger ones taken up both actively and passively	• 20 nm NPs cytotoxic to U937 and THP-1 cells • 20 nm NPs stimulated IL-8 secretion in human monocytes and induced measurable oxidative burst in monocytes • 500 and 1000 nm NPs stimulated IL-6 and IL-8 secretion in monocytes and macrophages, chemotaxis and phagocytosis of bacteria by macrophages, and provoked an oxidative burst of granulocytes • At lower concentrations with no cytotoxicity, 20 nm NPs inhibited, while 500 and 1000 nm NPs increased phagocytosis of bacteria by DMBM-2	Prietl et al., 2014 [115]
T98G (human glioblastoma cell line) HeLa (human cervical adenocarcinoma cell line)	PE microparticles (3–16 µm) PS particles (10 µm)		• Induced ROS generation • Cytotoxic effect, with PE having a higher EC_50_ value compared to PS in both T98G and HeLa cells	Schirinzi et al., 2017 [116]
Caco-2 (human epithelial colorectal adenocarcinoma cell line)	PS particles (0.1 and 5 µm)	Cellular uptake of nanoparticles	• Low toxicity on cell viability, oxidative stress, and membrane integrity and fluidity • Disruption of mitochondrial membrane potential • Inhibition of plasma membrane ATP-binding cassette (ABC) transporter activity	Wu et al., 2019 [117]
Human dermal fibroblasts Peripheral blood mononuclear cells (PBMCs) HMC-1 (human mast cell line 1) RBL-2H3 (human basophilic leukemia cell line) RAW 264.7 (mouse macrophage cell line)	PP particles (~20 µm and 25–200 µm), either first dispersed in DMSO or used directly in culture media		• Some degree of cytotoxicity at high dosages of the smaller size 20 µm particles • Low degree of induction of proinflammatory cytokines IL-6 and TNF-α from PBMCs • Increased histamine release from HMC-1 and RBL-2H3 cells • Some degree of ROS induction at high dosages of the smaller size 20 µm particles	Hwang et al., 2019 [118]
BEAS-2B (human lung epithelial cells)	PS MPs (4.06 ± 0.44 µm at 1–1000 μg/cm^2^		• Cytotoxic effects • Oxidative stress and inflammatory responses • Disruption of epithelial layer	Dong et al., 2020 [120]
A549 (Human alveolar type II epithelial cell line)	PS nanoparticles (25 and 70 nm)	Cellular uptake of nanoparticles	• Decreased viability and induced cell cycle arrest • Upregulation of transcripts for NF-κB and some pro-inflammatory cytokines • Alteration of cell cycle and apoptosis-regulation related protein expressions	Xu et al., 2019 [121]
BEAS-2B (Human bronchial epithelial cells)	PS nanoparticles	Cellular uptake of PS nanopaticles	• PS NPs only cytotoxic at very high concentrations • Metabolomics analyses revealed autophagic and endoplasmic reticulum (ER) stress-related metabolic changes	Lim et al., 2019 [122]
Hs27 (Human fibroblasts)	PS nanoparticles (100 nm at 5–75 µg/ml)		• Stimulation of ROS production • Genotoxic stress and DNA damage measured with the cytokinesis-block micronucleus (CBMN) assay	Poma et al., 2019 [119]
**No or Insignificant Effects**				
Caco-2	Polyethylene terephthalate (PET) NPs (laser ablated, ca. 100 nm)	Cellular uptake of NPs	• No apparent toxic effect • Nano-PET are internalized into endo-lysosomal compartments • Nano-PET has high propensity to cross the Caco-2 intestinal barrier model	Magri et al., 2018 [113]
Caco-2 THP-1 monocytic line	PS microparticles (1, 4, and 10 µm)	Cellular uptake of PS microparticles	• Low crossing of the cell monolayer on Transwells even by 1 µm microparticles • No pronounce loss of cell viability except only at very high dosage of 1 µm microparticles • Microparticles uptake did not affect macrophage differentiation or polarization	Stock et al., 2019 [109]
Caco-2 and HT29-MTX-E12 (human colon epithelial cell) co-culture BeWo b30 (Human placental trophoblast cell)	Carboxy-modified PS nanoparticles (50 nm and 0.5 μm,	Cellular uptake of PS nanoparticles	• No significant cytotoxicity unless at very high concentrations • No significant transport across the in vitro intestinal and placental “barriers” but intercellular distribution was observed	Hesler et al., 2019 [114]
